# Supplementary material for: Altered Regulation of the Glucose Transporter GLUT3 in PRDX1 Null Cells Caused Hypersensitivity to Arsenite
Source: Cells. 2023 Nov 22;12(23):2682. doi: 10.3390/cells12232682 (PMC10705171; doi:10.3390/cells12232682)
Supplement: Supplementary file 1 [file cells-12-02682-s001.zip › cells-2680635-supplementary.pdf]

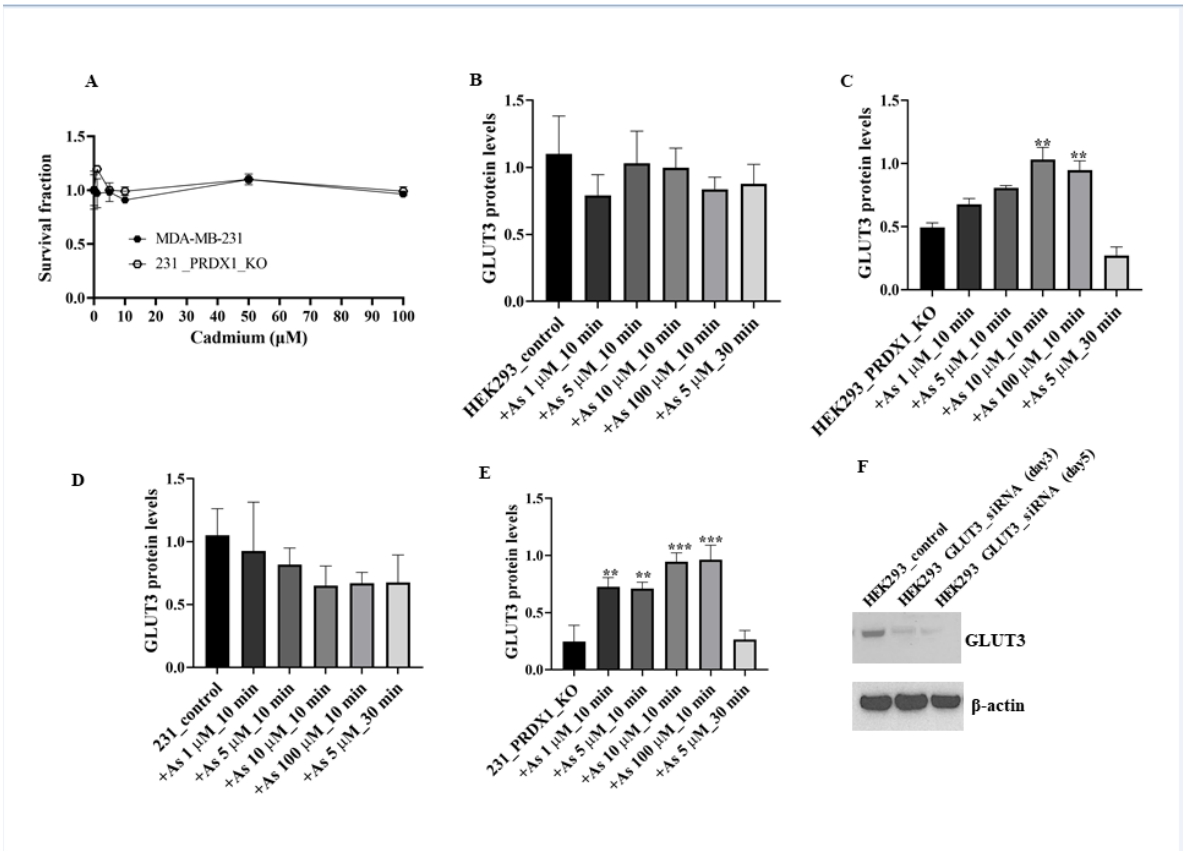

Figure S1. (**A**) Clonogenic survival assay showing cadmium sensitivity in MDA- MB-231 and MDA-MB-231 _PRDX1 null cells. (**B** and **C**) Quantification of GLUT3 protein levels by Western blot in HEK293_control and HEK293_PRDX1 null cells treated with the indicated concentrations of arsenite. (**D** and **E**) Quantification of GLUT3 protein levels by Western blot in MDA-MB-231_control and MDA-MB-231_PRDX1 null cells treated with the indicated concentrations of arsenite. (**F**) Western blot showing GLUT3 knockdown by siRNA in HEK293 cells. β-actin was used as a loading control.
